# Peritraumatic reactions in relation to trauma exposure and symptoms of posttraumatic stress in high school students

**DOI:** 10.1080/20008198.2017.1380998

**Published:** 2017-10-16

**Authors:** Nikolas Aho, Marie Proczkowska Björklund, Carl Göran Svedin

**Affiliations:** ^a^ Linköping University, Department of Clinical and Experimental Medicine, Child and Adolescent Psychiatry, Linköping, Sweden

**Keywords:** Trauma exposure, peritraumatic reactions, childhood trauma, PTSD, JVQ, TSCC, mediation, Exposición al trauma, reacciones peritraumáticas, trauma en la infancia, TEPT, JVQ, TSCC, mediación., 创伤暴露, 围创伤反应, 童年创伤, PTSD, JVQ, TSCC, 冥想, • From a large representative sample of Swedish adolescents, we found that peritraumatic reactions in general, and specifically peritraumatic dissociation, seem to explain the relationship between a traumatic event and symptoms. • Peritraumatic dissociation was more frequent in males; intervention thoughts were more common for females. • Attention to peritraumatic reactions seems warranted in clinical settings as further research will expand the knowledge in this area.

## Abstract

**Background**: Exposure to traumatic events is clearly associated with a diversity of subsequent mental health problems, with posttraumatic stress disorder (PTSD) as the most prevalent disorder. Epidemiologically, trauma exposure rates are more prevalent than PTSD, indicating that most trauma victims do not develop PTSD. More knowledge is needed to understand the development of the different posttraumatic pathways including the significance of pretraumatic, peritraumatic and posttraumatic risk factors.

**Objective**: To study peritraumatic reactions in relation to trauma exposure and symptoms of posttraumatic stress and to enhance our understanding of peritraumatic reactions as mediators between trauma and later symptomatology.

**Method**: The study was composed of a representative community sample of 5332 second year high school students (mean age 17.3 years) who completed the Juvenile Victimization Questionnaire (SAQ/JVQ), Trauma Symptom Checklist for Children (TSCC) and answered questions about peritraumatic reactions. Mediation effects of peritraumatic reactions on the trauma exposure relationship to symptoms was tested using the PROCESS macro for SPSS.

**Results**: Traumatic events are common (84.1%) and are accompanied in three-quarters of the students with at least one form of peritraumatic reaction. Peritraumatic reactions, especially peritraumatic dissociative reactions, mediate the relationship between trauma exposure and symptoms, and gender moderates the effect of peritraumatic dissociation. This moderating effect was found to be larger for boys than for girls, indicating gender differences in response to trauma.

**Conclusions**: The results indicate the need to screen for peritraumatic reactions as early as possible after a traumatic event in order to identify those at risk for PTSD.

## Abstract (es)

**Planteamiento**: La exposición a eventos traumáticos está claramente asociada con toda una variedad de problemas de salud mental posteriores, siendo el trastorno de estrés postraumático (TEPT) el trastorno más prevalente. Epidemiológicamente, las tasas de exposición al trauma son más frecuentes que el TEPT, lo que indica que la mayoría de las víctimas de trauma no desarrollan TEPT. Se necesita más conocimiento para comprender el desarrollo de las diferentes vías postraumáticas, incluidas la importancia de los factores de riesgo pretraumáticos, peritraumáticos y postraumáticos.

**Objetivo**: Estudiar las reacciones peritraumáticas en relación con la exposición al trauma y los síntomas de estrés postraumático y mejorar nuestra comprensión de las reacciones peritraumáticas como mediadores entre el trauma y la sintomatología posterior.

**Métodos**: El estudio se compuso de una muestra representativa de la comunidad de 5332 estudiantes de secundaria de segundo año (edad promedio 17,3 años) que completaron el Cuestionario de Victimización Juvenil (SAQ/JVQ, siglas en inglés de *Juvenile Victimization Questionnaire*), la Lista de Verificación de Síntomas de Trauma para Niños (TSCC, siglas en inglés de *Trauma Symptom Checklist for Children*) y respondieron preguntas sobre reacciones peritraumáticas. Los efectos mediadores de las reacciones peritraumáticas sobre la relación entre la exposición al trauma y los síntomas se probaron mediante el uso de la macro Hayes (2013) PROCESS SPSS.

**Resultados**: Los acontecimientos traumáticos son frecuentes (84,1%) y se acompañan en tres cuartas partes de los alumnos con al menos una forma de reacción peritraumática. Las reacciones peritraumáticas, especialmente las reacciones disociativas peritraumáticas, median la relación entre la exposición al trauma y los síntomas, y el género modera el efecto de la disociación peritraumática. Se encontró que este efecto moderador era mayor en los niños que en las niñas, lo que indica diferencias de género en respuesta al trauma.

**Conclusión**: Los resultados indican la necesidad de detectar reacciones peritraumáticas tan pronto como sea posible después de un acontecimiento traumático con el fin de identificar a los que corren riesgo de TEPT.

## Abstract (zh)

**背景：**创伤事件暴露和随后多种心理健康问题明确相关，其中创伤后应激障碍（PTSD）是最常见的。从流行病学上看，创伤暴露率要高于PTSD，说明大多数创伤受害者没有发生PTSD。还需要更多知识来了解不同的创伤后通路发展，包括创伤前、围创伤期和创伤后危险因素。

目标：研究围创伤反应与创伤暴露和创伤后应激症状的关系，并增加对围创伤反应对创伤和随后症状之间关系的调节作用。

**方法：**本研究使用一个包含5332名二年级高中生的代表性社区样本（平均年龄17.3岁），被试完成了青少年侵害问卷（SAQ/JVQ）和儿童版创伤症状检查清单（TSCC），并回答了关于围创伤反应的问题。使用Hayes（2013）PROCESS SPSS宏检查围创伤反应对创伤暴露和症状的中介作用。

**结果：**创伤性事件是常见的（84.1%），在3/4学生中同时出现至少一种形式的围创伤反应。围创伤反应，特别是围创伤分离反应，中介了创伤暴露和症状之间的关系；性别调节围创伤分离反应的作用。这种调节效应在男生中强于女生，说明对创伤的反应存在性别差异。

**结论：**结果说明需要在创伤事件后尽快筛选围创伤反应以识别具有PTSD风险的个人。

Childhood trauma exposure, especially in the form of child physical and sexual abuse, has since the mid-1990s merited increased attention from media as well as from researchers and policy makers (Zwi, Krug, Mercy, & Dahlberg, 2002). This has resulted in a series of international protocols (Butchart, World Health Organization, & International Society for the Prevention of Child Abuse and Neglect, 2006; Council of Europe, 2012; United Nations Office of the High Commissioner for Human Rights, 2000), including the Global Partnership to End Violence against Children, which has set a goal of ending all forms of violence against children by 2030 as part of the UN Sustainable Development Goals (United Nations Division for Sustainable Development, 2015).

Childhood trauma exposure is prevalent. The US Children’s Bureau reported a rate of 9.4 victims per 1000 children in the 2014 population, of which 75.0% were victims of neglect, 17.0% were physically abused, and 8.3% sexually abused (U.S. Department of Health & Human Services, 2016). The same report stated that 1580 children died of abuse and neglect, corresponding to a rate of 2.13 per 100,000 children in the US population (U.S. Department of Health & Human Services, 2016). Studies using extensive standardized questionnaires with representative samples show that 71% (US) and 84% (Sweden) of the participating children under the age of 18 reported at least one type of violence exposure (Aho, Proczkowska-Bjorklund, & Svedin, 2016; Finkelhor, Turner, Ormrod, & Hamby, 2009). Victimization is more prevalent among women in all categories except for conventional crime (Aho, Gren-Landell, & Svedin, 2016), and females experience more events than males with the exception of conventional crime (Aho et al., 2016).

Several health effects have been reported in relation to trauma exposure. In their meta study, Hawker and Boulton (2000) found associations between trauma exposure and psychosocial maladjustment, including depression, loneliness, global low self-esteem, poor social self-concept, generalized anxiety and social anxiety, with effect sizes (Pearson’s *r*) ranging from .25 to .45. Chan (2013) reported that child victims were more likely to report posttraumatic stress disorder (PTSD), depressive symptoms, self-harm ideation and poor physical or mental health. Takizawa, Maughan, and Arseneault (2014) concluded that victimized individuals were at risk for a wide range of poor social, health and economic outcomes nearly four decades after exposure.

Although multiple findings support the conclusion that trauma exposure is harmful, interpersonal events (one-on-one interactions) are found to have greater weight than non-interpersonal events regarding PTSD symptoms. This effect is larger among female adolescents than among males, indicating a developmental gender difference regarding vulnerability (Gustafsson, Nilsson, & Svedin, 2009). Female gender and interpersonal events are also more related to complex PTSD in children and adolescents (Sachser, Keller, & Goldbeck, 2017).

Of the theoretical diathesis-stress models proposed to identify vulnerability factors or predictors of PTSD development, the most recent models propose that pretrauma individual risk factors (diatheses) contribute to a constitutional vulnerability to a situational stressor/trauma (Bomyea, Risbrough, & Lang, 2012; Elwood, Hahn, Olatunji, & Williams, 2009; McKeever & Huff, 2003). This stressor must be sufficiently severe to activate the diathesis and promote the development of PTSD. According to Elwood et al. (2009), comprehensive diathesis stress model of PTSD should take into account not only pretrauma factors but also peritrauma and posttrauma factors, as well as different types of vulnerability (e.g. biological, psychological, cognitive). Pretrauma factors can include age, gender, race or ethnicity, education, prior psychopathology and neurobiological factors. Peritrauma factors can include the duration or severity of trauma experience and the perception that the trauma has ended. Posttrauma factors can include access to needed resources, social support, specific cognitive patterns and physical activity (Sayed, Iacoviello, & Charney, 2015).

There has been an increasing interest in understanding the role of peritraumatic variables (i.e. physiology, affect, cognition) that occur during the trauma (Bernat, Ronfeldt, Calhoun, & Arias, 1998). Attention has been paid to the construct of peritraumatic dissociation (PD) (i.e. dissociation that occurs during the event: experiencing moments of losing track or blanking out, having an altered sense of time, feeling as if one is floating above the scene, feeling disconnected from one’s body) (Marmar et al., 1994). A proliferation of related research has been published since the middle of the 1990s (Ehlers, Mayou, & Bryant, 1998; Griffin, Resick, & Mechanic, 1997; Koopman, Classen, & Spiegel, 1994; Marmar, Weiss, Metzler, & Delucchi, 1996; Shalev, Peri, Canetti, & Schreiber, 1996). Several studies have provided evidence for an association between PD and symptoms of PTSD (Boelen, 2015; Boelen, Keijsers, & Van Den Hout, 2012; Bui et al., 2013). These studies found that the impact of violent loss and the unexpectedness of the loss on PTSD severity was fully mediated by peritraumatic distress and dissociation; peritraumatic helplessness and PD emerged as unique mediators. Prospective studies of women found peritraumatic distress predictive of acute PTSD, defined as one month after the traumatic event, whereas PD predicted mid-term PTSD at four months posttrauma (Gandubert et al., 2016; Johnson, Pike, & Chard, 2001). These studies also found that, in adult female CSA victims, PD was the only variable found to significantly predict symptom severity across symptom type or disorder.

The primary objective of the current paper was to study peritraumatic reactions in relation to trauma exposure and symptoms of posttraumatic stress in a large representative sample of adolescents in Sweden. The current study also seeks to enhance our understanding of peritraumatic reactions as mediators between trauma and later symptomatology. This paper is part of a larger ongoing project studying trauma exposure and child health in relation to the contribution of genetic and psychosocial background to the risk for development of posttraumatic disorders and other clinical and subclinical conditions during adolescence.

## 1. Method

### 1.1. Participants

Municipalities were chosen out of nine municipality categories developed by the Swedish Association of Local Authorities and Regions (SALAR, 2009). Schools were selected from registers of The Swedish National Agency for Education (2007) to represent the national average concerning gender, birthplace, enrolment from various municipalities and educational programmes, and to include a proportion of students in the introductory programme. One municipality category, ‘sparsely populated municipalities’ (SKL 5), was omitted due to lack of high schools. All schools were public schools except for some private schools in the SKL 3 category.

A total of 53 schools were asked to participate in the survey. Two schools declined to participate. The 51 participating schools enrolled a total of 7849 second year students. A total of 6096 students (78%) were present at the scheduled survey. The missing students were absent by plan or absent without notice (skipping class). Out of the 6096 students present, 136 were not willing or able to complete the survey, resulting in an external attrition of 22% and an internal attrition of 2.2%. A tentative analysis suggests that the dropout group might have lower socioeconomic status and that trauma exposure might be more prevalent in this group. Due to technical failure, a total of 628 cases were lost for JVQ item #3 and JVQ item #5. Cases with missing items were removed, resulting in a total of 5332 cases.

The sample included 5332 second year high school students, with a mean age of 17.3 (range 16–20 years of age *SD* 0.659). The sample correspond well with the national population distribution among municipality categories, with ±10% variation from the national average. The sample was merged from nine into three municipality categories, with 19.1% of the students from large municipalities (>200,000 inhabitants), 41.8% from medium municipalities (50–200,000 inhabitants) and 39.1% from small municipalities (<50,000 inhabitants). Of the sample, 51.4% were young men and 48.6% were young women. The majority of socio-demographic variables were in line with population measures. Two measures deviated: residing with both parents was more frequent, and parents had higher unemployment rates. For socio-demographic data concerning adolescent birthplace, parents’ birthplace and employment, residence and educational programme (see Table 1).

**Table 1.** Sociodemographic background data.

	Sample		Reference Group
	%	*n*	%
Gender			
Male	51.4	2741	51.6
Female	48.6	2591	48.4
Birthplace Adolescent			
Sweden	91.4	4871	91.0
Europe	4.1	220	2.4
Outside Europe	4.5	241	6.6
Birthplace Parents			
Both parents born in Sweden	78.4	4178	81.5^a^
One or both in Europe	8.7	464	18.0^a^
One or both outside Europe	12.9	690	_
Employment Parents			
Both employed	68.3	3641	87.3^b^
At least one unemployed^c^	31.7	1691	12.7
Residency			
Both parents^d^	74.5	3973	59.2
One parent	20.3	1080	39.9
Alone or other	5.2	279	0.9
Education Programme			
Theoretical	41.4	2206	34.6^e^
Practical	56.9	3033	55.7^e^
Introductory	1.7	93	9.8^e^
Municipality Size (inhabitants)			
Large>200,000	19.1	1018	21.2^f^
Medium 50,000–200,000	41.8	2229	46.6^f^
Small <50,000	39.1	2085	32.2^f^

Statistics Sweden registers contain data of the population by sex, age, marital status, country of birth and citizenship for all of Sweden, in each county, in county-blocks, metropolitan areas and each municipality. Asylum seekers who have not yet obtained a permit to stay are not included in the population statistics. In 2008, there was a total population of 9,256,347: male = 4,603,710; female = 4,652,637. Total number of 17-year-olds was 131,366: male = 67,836; female = 63,530. The Swedish National Agency for education report total second grade students at 83,953: male = 42,097; female = 41,856. *N* = 5332.

^a^Statistics Sweden, both within and outside Europe, biological born abroad or adopted abroad excluded.

^b^Statistics Sweden, Unemployment 15 to 74 years.

^c^Unemployment = unemployed, being a student, on parental leave, not working due to disability or chronic or temporary illness.

^d^Living with both parents of origin or alternate residence.

^e^The Swedish National Agency for Education.

^f^Statistics Sweden, three categories made from nine SCB categories.

## 2. Research procedure

A standardized information letter was sent to 51 schools following the initial contact and request to conduct the survey. Participating schools were asked to set up a suitable room for the survey, arrange a schedule and appoint a teacher responsible for each class. Student attendance was noted on the register. All students were initially handed one page of written information about the project and contact information to use in the event that they felt any discomfort answering the questions that were asked. Prior to data collection the students received written information about the study and gave informed consent for participation in the survey. According to the Ethical Review Act of Sweden (2003), active consent is not required from parents when adolescents are 15 years of age or older.

The survey was administered on PCs provided by the school or the researcher, and the researcher was present for information and for answering any questions. None of the items could be omitted, limiting internal attrition on the item level. The students completed the survey in 30–40 minutes and were given movie vouchers.

## 3. Questionnaires

The composite questionnaire consisted of introductory questions: location of survey, gender, birthplace, age, educational programme, parents’ birthplace, parents’ employment, and residence, followed by five standardized questionnaires, two of which are included in this paper: The Self-Administered Juvenile Victimization Questionnaire (SAQ/JVQ) and the Trauma Symptom Checklist for Children (TSCC). Specific questions regarding peritraumatic reactions concerning the latest reported trauma exposure event were added to the JVQ. The four final questions concerned debut age and consumption level for alcohol, sexual debut age, contact with professionals such as school psychologist, school counsellor, social worker, child psychiatrist or representatives of BRIS (BRIS, or Children´s Rights in Society, is a politically and religiously independent children’s rights organization that listens to, supports and strengthens children and young adults’ rights in society) and history of medication for mood disorder, hyperactivity or trouble sleeping. Only the introductory questions from the SAQ/JVQ, the TSCC and the questions about different peritraumatic reactions are presented in this paper.

## 4. The Juvenile Victimization Questionnaire (SAQ/JVQ)

The JVQ was designed to be a more comprehensive instrument than questionnaires used in previous research, providing an inventory of most of the major forms of offense against young people, including nonviolent victimization and events not typically conceptualized as crimes (Finkelhor, Ormrod, Turner, & Hamby, 2005). The JVQ obtains reports on 34 forms of offense against young people that cover five general domains of concern. For the purpose of the current research, a modified self-administered version of the questionnaire (SAQ) was used. One survey item concerning sexual victimization which asked, ‘Did you do sexual things with anyone 18 years or older, even things you both wanted?’ was excluded due to differences between the legal systems in the USA and Sweden, resulting in a total of 33 JVQ items. The JVQ covers victimizing events during the prior year (PY) and before the prior year (BPY), which also makes it possible to assess lifetime (LT) events. If victimization occurred in the PY and/or BPY, the participant was instructed to answer follow-up questions about the last event regarding peritraumatic reactions. The JVQ has been tested for construct validity and reliability with good results. (Finkelhor, Hamby, Ormrod, & Turner, 2005). The event items fall within five domains: conventional crime trauma exposure (items 1–8), childhood maltreatment (items 9–12), peer and sibling trauma exposure (items 13–18), sexual trauma exposure (items 19–24, excluding the item statutory rape) and witnessing and indirect trauma exposure (items 26–34). In this study, the internal consistency (Cronbach´s alpha) was calculated for the full JVQ scale α = 0.83 and for conventional crime α = 0.66, childhood maltreatment α = 0.55, peer and sibling victimization α = 0.52, sexual victimization α = 0.64, and witnessing and indirect victimization α = 0.51.

## 5. The Trauma Symptom Checklist for Children (TSCC)

The TSCC (Briere, 1996) is a self-report questionnaire about trauma related symptoms. It consists of 54 items (scored 0–3), six main clinical subscales (anxiety, depression, anger, posttraumatic stress, dissociation, sexual concerns) and two validity scales (hyper-response, under-response). The clinical scales are added up to give a total score. The Swedish translation of the questionnaire has displayed satisfactory psychometric properties for Swedish adolescents (Nilsson, Wadsby, & Svedin, 2008). The total score (TSCC tot) and the subscales (TSCC dis and TSCC pts) were used as the main measures of health. Cronbach’s alpha was 0.96, 0.88 and 0.85 for TSCC tot, TSCC pts and TSCC dis, respectively (based on cases with JVQ events >0).

## 6. Peritraumatic reactions

To assess peritraumatic reactions of physiological arousal (PA), dissociation and intervention thoughts (IT), three scales developed by Dyb et al. (2008) were used. All items in these scales were dichotomous 0/1. Three items described physiological reactions at the time of the trauma, including increased heart and respiration rate and perspiration; these items formed the PA scale (range 0–3), α = 0.90. A five-item scale was developed to capture reports of dissociation at the time of the traumatic experience, known as PD, which includes experiences such as derealization, depersonalization and alterations in perception of time or place. PD subscale scores ranged from 0–5, α = 0.90. Three items described IT at the time of the trauma, including thoughts of altering the precipitating events and interruption of the traumatic action by self or others. IT subscale scores ranged from 0–3, α = 0.89.

## 7. Statistical analyses

The prevalence rates were reported as lifetime. Missing cases were not analysed. Trauma was measured by total life events according to the JVQ. Trauma symptoms were measured by three TSCC variables (total, posttraumatic, dissociation) and four variables of PT reactions (total, PA, PD, IT).

Correlation is reported as Spearman rho. To test whether peritraumatic reactions had an indirect mediating effect on the relationship between traumatic events and symptoms, multiple regression analyses were performed using Hayes (2013) PROCESS SPSS macro, which allows for the testing of models with bias corrected and accelerated confidence interval (BCa CI). In mediation models, Path A indicates the direct association between the independent variable and mediator, Path B indicates the direct association between the mediator and dependent variable, Path C represents the association between the independent and dependent variables without the inclusion of the mediator, and Path C’ represents the direct effect of the independent on the dependent variable holding the mediator constant. In bootstrapped models a significant indirect effect, as evidenced by the exclusion of 0 from the 95% CI, is consistent with statistical mediation. P-values were used to test significance for the other paths. Mediation was analysed following instructions from Field (2013; Hayes, 2013).

First, we built a model of the lifetime number of victimizing events, with the JVQ as the independent variable, and trauma symptoms, with the TSCC sum as the dependent variable. Second, we included the PT tot as mediators in the model, followed by a model where the different PT subscales were differentiated in a parallel fashion, with the JVQ as the independent variable and the TSCC sum as the dependent variable. Since gender has been known to influence the development of symptoms after trauma and because different types of trauma can be seen in boys and girls, two different models were analysed in the mediating analysis. All analyses were performed IBM SPSS Statistics version 24.0.

## 8. Results

### 8.1. Descriptive statistics

Of the *N* = 5332 cases, a total of *n* = 4483 (84.1%) reported at least one victimizing lifetime event (males = 83.0%, females = 85.2%). Out of these, 74.9% (*n* = 3360) also experienced a PT reaction of some kind: PA was reported by 31.9% (*n* = 1428), PD was reported by 48.2% (*n* = 2159) and IT was reported by 63.2% (*n* = 2834). Table 2 shows univariate analyses of peritraumatic reactions and TSCC.

**Table 2.** Descriptive data of study variables.

Variable	Mean	Range	*SD*
JVQ (lifetime events)	4.12	33	4.043
Peritraumatic reactions			
Total (PT)	4.94	152	9.942
Physiological arousal (PA)	1.06	47	2.990
Peritraumatic dissociation (PD)	1.56	55	3.774
Intervention thoughts (IT)	2.32	76	4.400
TSCC			
Total	29.15	162	21.279
Dissociation	5.77	30	4.635
Posttraumatic stress	6.27	30	5.190

*N* = 5332

As shown in Table 3, traumatic events (JVQ) correlated with peritraumatic reactions and revealed moderate to strong correlations with PT total (*r*s = 0.73, *p* > .001), PA (*r*s = .50, *p* < .001), PD (*r*s = .59, *p* < .001) and IT (*r*s = .65, *p* < .001). Traumatic events and posttraumatic symptoms correlated weakly to moderately, but significantly. All of the different peritraumatic reactions correlated weakly with all of the different trauma symptoms endpoints (see Table 3).

**Table 3.** Bivariate, zero order correlation of events, peritraumatic reactions and symptoms.

		Peritraumatic reactions				Trauma symptoms (TSCC)		
	JVQ	Total	PA	PD	IT	Total	Dis	PTS
JVQ	1							
Peritraumatic reactions total (PT)	0.73	1						
Physiological arousal (PA)	0.50	0.68	1					
Peritraumatic dissociation (PD)	0.59	0.79	0.52	1				
Intervention thoughts (IT)	0.65	0.90	0.51	0.60	1			
TSCC total	0.41	0.41	0.29	0.33	0.38	1		
TSCC dissociation	0.34	0.35	0.25	0.28	0.33	0.87	1	
TSCC posttraumatic stress	0.37	0.40	0.28	0.33	0.38	0.87	0.75	1

All values significant. *p* < .001. Spearman rho (two tailed). *N* = 5332.

### 8.2. Mediation analysis with PT total

**Figure 1.**
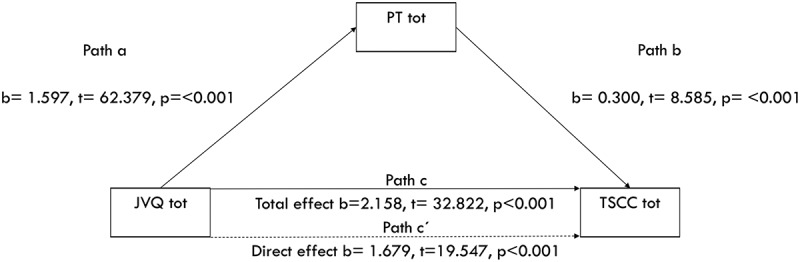
Mediation of the association between trauma exposure and trauma symptoms by peritraumatic reactions. Unstandardized B coefficients are displayed with t and *p* values. Trauma exposure indicates total trauma exposure measured by the JVQ lifetime total scale, trauma symptoms indicates total score from the TSCC, peritraumatic reactions are measured by peritrauma total scale. Path c indicates the association between trauma exposure and trauma symptoms without the inclusion of a mediator. Path c´ indicates the direct effect, i.e. controlling for the mediator in the model. Paths a, b and c are significant as evidence by *p* ≤ .01. The ‘t’ statistic is computed by dividing the estimated value of the parameter by its standard error. This statistic is a measure of the likelihood that the actual value of the parameter is not zero. The larger the absolute value of t, the less likely that the actual value of the parameter could be zero.

The JVQ positively predicts the TSCC tot (path c), b = 2.158, t = 32.822, *p* ≤ .001 (see Figure 1). This is the total effect of the JVQ on the TSCC sum without controlling for any mediators. Looking at the mediating effect of PT tot on the TSCC tot, the JVQ significantly predicts PT tot (path a), b = 1.597, t = 62.379, *p* ≤ .001 and 42.2% of the PT tot variance is explained. PT tot significantly predicts the TSCC tot (path b), b = 0.300, t = 8.585, *p* ≤ .001 with 17.9% of the TSCC sum variance explained. The direct effect of the JVQ on the TSCC tot is b = 1.679, t = 19.547, *p* < .001 and the indirect effect mediated by PT tot is b = 0.479, BCa CI [0.342 – 0.640], representing a relatively small effect of 7.6%, κ2 = 0.076, 95% BCa CI [0.054–0.101]. The model explains 18% of the variance.

### 8.3. Mediation with peritraumatic reactions: PA, PD and IT

**Figure 2.**
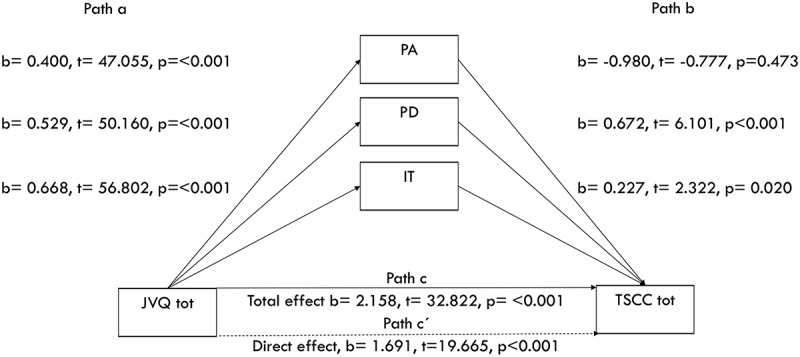
Mediation of the association between trauma exposure and trauma symptoms by peritraumatic reactions. Unstandardized B coefficients are displayed with t and *p* values. Trauma exposure indicates total trauma exposure measured by the JVQ lifetime total scale, trauma symptoms indicates total score from the TSCC, peritraumatic reactions are measured by peritrauma scales. Path c indicates the association between trauma exposure and trauma symptoms without the inclusion of a mediator. Path c´ indicates the direct effect, i.e. with the mediator. Paths a, b and c´ are significant as evidence by *p* ≤ .01. The ‘t’ statistic is computed by dividing the estimated value of the parameter by its standard error. This statistic is a measure of the likelihood that the actual value of the parameter is not zero. The larger the absolute value of t, the less likely that the actual value of the parameter could be zero.

The JVQ predicts the pathways (path a) to the different peritraumatic reactions: PA b = 0.400, t = 47.055, *p* < .001, explaining 29% of the variance, PD b = 0.529, t = 50.160, *p* < .001, explaining 32% of the variance and IT b = 0.668, t = 56.802, *p* < .001, explaining 37% of the variance (see Figure 2). The different PT reactions PD and IT significantly predict the total score on TSCC (path b) in the model; PA: b = −0.980, t = −0.777, *p* = .473, PD: b = 0.672, t = 6.101, *p* ≤ .001 and IT: b = 0.227, t = 2.322, *p* = .020. The indirect effect of the JVQ on the TSCC tot mediated by the different PT reactions is only significant regarding the PD (b = 0.355, BCa CI [0.199–0.523]), since the other two reactions’ confidence interval included 0.

Inspection of the conditional indirect effects at different levels of the moderator (males versus females) shows that the model explains more of the variance on the TSCC tot for females (females 21.7%, males 11.7%) (see Table 4). There was a stronger mediation effect of PD between the JVQ and the TSCC for males (b = 0.394 BCa CI [0.170–0.636]) than for females (b = 0.247, BCa CI [0.021–0.469]).

**Table 4.** Separated mediating models for males/females with TSCC tot, TSCC dis and TSCC pts as dependent variables and JVQ as independent variable. First model with PT total, then separating the different mediating factors PA, PD and IT.

TSCC							
		Tot		Dis		PTS	
Independent		b	BCa CI	b	BCa CI	b	BCa CI
PT total	F	0.35	0.16–0.53	0.06	0.02–0.12	0.12	0.08–0.17
	M	0.32	0.12–0.56	0.07	0.02–0.11	0.10	0.05–0.16
PA	F	0.28	−0.18–0.20	0.00	−0.04–0.04	0.00	−0.05–0.05
	M	−0.07	−0.30–0.13	−0.02	−0.06–0.02	0.00	−0.05–0.04
PD	F	0.23	0.02–0.50	0.05	−0.00–0.10	0.07	0.01–0.12
	M	0.43	0.20–0.68	0.09	0.04–0.14	0.09	0.05–0.14
IT	F	0.09	−0.14–0.32	0.01	−0.04–0.07	0.06	0.01–0.11
	M	−0.90	−23.00–0.00	−0.02	−0.04–0.00	−0.01	−0.03–0.02

# BCa CI = Corrected and Accelerated Confidence Interval. Significance is obtained when 0 is not in the confidence interval. Mediation analysis was performed with the use of the Process macro for SPSS by Andrew F. Hayes.

Different mediating models for males and females were tested with TSCC dis and TSCC pts as dependent variables parallel with the TSCC tot (see Table 4). PA did not have any significant indirect effect in any of the models. In males, a significant indirect mediating effect of PD could be seen in the different models, while PD in females showed a significant effect regarding TSCC total and TSCC pts. IT did not show any indirect effect in males, but showed a significant effect for females regarding TSCC pts.

## 9. Discussion

This study investigated the relationship between peritraumatic reactions, trauma exposure and symptoms of posttraumatic stress, and the relationship between trauma and posttraumatic symptoms mediated by peritraumatic reactions, among adolescents in Sweden. The main results from this study can be summarized in three findings.

First, traumatic experiences are common among young people (84.1%), and these experiences are accompanied in three-quarters of those with trauma experiences by at least one form of peritraumatic reaction, most commonly IT.

Second, the study confirms that peritraumatic reactions were related to the TSCC (b = 0.300, t = 8.585, *p* ≤ .001), found in adults (Briere, Scott, & Weathers, 2005) and youth (Dyb et al., 2008). Separating the different peritraumatic reactions for males and females, PA did not show any significant indirect effect, while PD showed a clear indirect effect for males and a more unclear picture for females. IT showed an indirect effect for females but not for males. The reason PA did not show any significant indirect mediating effect may be that PA reactions (increased heart rate, faster breathing, sweating) commonly result from physical exertion, and may not necessarily be responses to stressful situations. As such, PA may have a more moderating role in the development of symptoms after trauma. The differences in the roles PD and IT play for males and for females may reflect gender differences in the process of symptom development after trauma, but also may be because trauma takes different forms for males and females since they have different developmental paths. These differences are in line with the study on Yezidi refugees in Turkey (Tekin et al., 2016), where men reported more overmodulation (dissociation) and women reported more intrusive thoughts (undermodulation). Both of these findings may reflect differences in social factors such as gender roles or differences in biological factors (sex) in relation to extreme stress. Finally, this finding supports the creation of a dissociative subtype of PTSD as suggested by, for example, Steuwe, Lanius, and Frewen (2012), however further research is needed.

Third, we found that PD at the time of the event was related to trauma symptoms (b = 0.769, t = 6.101, *p* < .001), as was also found in a normal sample of children by Dyb et al. (2008) and in a clinical sample of youth by Sugar and Ford (2012). The association of PD and current PTSD reactions is also consistent with findings of studies of PTSD in adults (Johnson et al., 2001; Lawyer et al., 2006; Marmar et al., 1994; Werner & Griffin, 2012).

In our model we found support for PD mediating the relationship between trauma and symptoms. Other researchers have obtained similar results, such as Bryant et al. (2011), who found peritraumatic dissociative mediation effects on the relationship between panic and PTSD symptoms among physical trauma patients. In the models, PA was not significant and IT was significant *p* < .05 (*p* = .035) with a relatively low b value (b = 0.277), with the conclusion that only PD acted as a mediator of the three variables tested. Similar results were reported by Dyb et al. (2008).

The mediating effect of dissociation was higher for males than for females. Similar results are reported by Bennett, Modrowski, Kerig, and Chaplo (2015) who found that PD mediated persistent dissociation only in males. Some studies have found higher rates of dissociation in girls than in boys in samples exposed to community violence (Zona & Milan, 2011).

A question for further research is whether gender differences in dissociation may depend on the type of trauma youth experience (Hetzel-Riggin & Roby, 2013). For example, girls are more likely than boys to experience traumatic events that are highly related to dissociation, such as sexual abuse (Bernier, Hebert, & Collin-Vezina, 2013; Collin-Vezina, Daigneault, & Hebert, 2013; Trickett, Noll, & Putnam, 2011). In the current study, we did not examine results by trauma type.

## 10. Strengths and limitations

The strength of this paper was the use of clearly defined questions covering a broad range of trauma exposures and psychiatric symptoms, a large sample generalizable to the population and a standardized recruitment process. No cause and effect relationship can be inferred due to the cross-sectional design. Because trauma exposure is not a randomly assigned condition it is not possible to state that the symptoms are caused only by the trauma exposure. We used lifetime data, which has an increased potential for attribution error and puts a strain on memory recall, leading to the possibility of biased results. Due to our data collection method, we could not use the separate incident version of the JVQ, with the effect that all trauma exposures counted equally regardless of whether they originated from the same incident or not, with the obvious result of a higher number of reported trauma exposure events. Due to the design of the survey we did not have temporal data; the student was asked about the most recent event when answering questions about reactions related to the event. In designing the survey, we chose to study the most recent event as this limits recall bias. We did not control for pretrauma dissociative tendencies, acute posttraumatic reactions, personality traits, social support, locus of control, initial depression, anxiety or prior PTSD, which are constructs with potential impact on posttrauma psychopathology (Engelhard, Van Den Hout, Kindt, Arntz, & Schouten, 2003; Ozer, Best, Lipsey, & Weiss, 2003). In this paper we chose to limit the research to the total scale of trauma exposure. Regarding the TSCC, we used the total scale as well as measures of dissociation and posttrauma symptoms, as these measures have been used by other researchers. However, the survey data may be analysed according to other domains and subscales within the TSCC. For items within the JVQ and peritraumatic events, further research will have to be done.

Nonparametric tests were used for bivariate analyses. No transformations were performed. Missing cases were deleted from the dataset. Outliers are known to affect results in regression analysis. We chose not to remove outliers, as we know from prior studies that some cases can have extremely high levels of trauma exposure. Because trauma questions and peritraumatic items are of score type and their distributions were positive, with a majority of cases assembled around zero, nonparametric tests were indicated. However, we chose to use parametric tests such as regression analyses as the sample was large and the dependent variable was normally distributed.

In this paper we chose to limit the research to total scale of trauma exposure. In this study we used data from the most recently reported events, but it would be important for future research to focus on the age of first exposure to adverse events because the relationship between total exposure to violence and symptoms of hyperarousal has been shown to be stronger for those first exposed at earlier ages (Miller-Graff, Scrafford, & Rice, 2016).

## 11. Conclusion

We found that symptoms after trauma were mediated by peritraumatic reactions, and that PD seems to have a clear role, especially for males. Identifying peritraumatic reactions as early as possible after the event may help to identify those at risk for PTSD and other psychiatric symptoms. It may also open the way for other possible treatments.

## References

[CIT0001] Aho N., Gren-Landell M., & Svedin C. G. (2016). The prevalence of potentially victimizing events, poly-victimization, and its association to sociodemographic factors: A Swedish youth survey. *Journal of Interpersonal Violence*, 31(4), 620–11. 25392393 10.1177/0886260514556105

[CIT0002] Aho N., Proczkowska-Bjorklund M., & Svedin C. G. (2016). Victimization, polyvictimization, and health in Swedish adolescents. *Adolescent Health, Medicine and Therapeutics*, 7, 89–99. 10.2147/AHMT.S109587 PMC5008259 27616895

[CIT0003] Bennett D. C., Modrowski C. A., Kerig P. K., & Chaplo S. D. (2015). Investigating the dissociative subtype of posttraumatic stress disorder in a sample of traumatized detained youth. *Psychological Trauma: Theory, Research, Practice and Policy*, 7(5), 465–472. 10.1037/tra0000057 26010107

[CIT0004] Bernat J. A., Ronfeldt H. M., Calhoun K. S., & Arias I. (1998). Prevalence of traumatic events and peritraumatic predictors of posttraumatic stress symptoms in a nonclinical sample of college students. *Journal of Traumatic Stress*, 11(4), 645–664. 9870219 10.1023/A:1024485130934

[CIT0005] Bernier M. J., Hebert M., & Collin-Vezina D. (2013). Dissociative symptoms over a year in a sample of sexually abused children. *Journal of Trauma and Dissociation*, 14(4), 455–472. 23796175 10.1080/15299732.2013.769478

[CIT0006] Boelen P. A. (2015). Peritraumatic distress and dissociation in prolonged grief and posttraumatic stress following violent and unexpected deaths. *Journal of Trauma and Dissociation*, 16(5), 541–550. 26156555 10.1080/15299732.2015.1027841

[CIT0007] Boelen P. A., Keijsers L., & Van Den Hout M. A. (2012). Peritraumatic dissociation after loss: Latent structure and associations with psychopathology. *Journal of Nervous and Mental Disease*, 200(4), 362–364. 22456592 10.1097/NMD.0b013e31824cc60e

[CIT0008] Bomyea J., Risbrough V., & Lang A. J. (2012). A consideration of select pre-trauma factors as key vulnerabilities in PTSD. *Clinical Psychology Review*, 32(7), 630–641. 22917742 10.1016/j.cpr.2012.06.008 PMC3444146

[CIT0009] Briere J. (1996). *Trauma symptom checklist for children (tscc), professional manual*. Odessa, FL: Psychological Assessment Resources.

[CIT0010] Briere J., Scott C., & Weathers F. (2005). Peritraumatic and persistent dissociation in the presumed etiology of PTSD. *American Journal of Psychiatry*, 162(12), 2295–2301. 16330593 10.1176/appi.ajp.162.12.2295

[CIT0011] Bryant R. A., Brooks R., Silove D., Creamer M., O’Donnell M., & McFarlane A. C. (2011). Peritraumatic dissociation mediates the relationship between acute panic and chronic posttraumatic stress disorder. *Behaviour Research and Therapy*, 49(5), 346–351. 21457945 10.1016/j.brat.2011.03.003

[CIT0012] Bui E., Simon N. M., Robinaugh D. J., Leblanc N. J., Wang Y., Skritskaya N. A., … Shear M. K. (2013). Periloss dissociation, symptom severity, and treatment response in complicated grief. *Depression and Anxiety*, 30(2), 123–128. 23212730 10.1002/da.22029 PMC3967786

[CIT0013] Butchart A., World Health Organization, & International Society for the Prevention of Child Abuse and Neglect (2006). *Preventing child maltreatment: A guide to taking action and generating evidence*. Geneva: World Health Organization.

[CIT0014] Chan K. L. (2013). Victimization and poly-victimization among school-aged Chinese adolescents: Prevalence and associations with health. *Preventive Medicine*, 56(3–4), 207–210. 23295171 10.1016/j.ypmed.2012.12.018

[CIT0015] Collin-Vezina D., Daigneault I., & Hebert M. (2013). Lessons learned from child sexual abuse research: Prevalence, outcomes, and preventive strategies. *Child and Adolescent Psychiatry and Mental Health*, 7(1), 22. 23866106 10.1186/1753-2000-7-22 PMC3720272

[CIT0016] Council of Europe (2012). *Protection of children against sexual exploitation and sexual abuse*. Strasbourg: Author.

[CIT0017] Dyb G., Rodriguez N., Brymer M. J., Saltzman W. R., Steinberg A. M., & Pynoos R. S. (2008). Emotional reactions, peritraumatic dissociation, and posttraumatic stress reactions in adolescents. *Journal of Child & Adolescent Trauma*, 1(1), 63–74.

[CIT0018] Ehlers A., Mayou R. A., & Bryant B. (1998). Psychological predictors of chronic posttraumatic stress disorder after motor vehicle accidents. *Journal of Abnormal Psychology*, 107(3), 508–519. 9715585 10.1037//0021-843x.107.3.508

[CIT0019] Elwood L. S., Hahn K. S., Olatunji B. O., & Williams N. L. (2009). Cognitive vulnerabilities to the development of PTSD: A review of four vulnerabilities and the proposal of an integrative vulnerability model. *Clinical Psychology Review*, 29(1), 87–100. 19008028 10.1016/j.cpr.2008.10.002

[CIT0020] Engelhard I. M., van den Hout M. A., Kindt M., Arntz A., & Schouten E. (2003). Peritraumatic dissociation and posttraumatic stress after pregnancy loss: A prospective study. *Behaviour Research and Therapy*, 41(1), 67–78. 12488120 10.1016/s0005-7967(01)00130-9

[CIT0021] Field A. (2013). *Discovering statistics using IBM SPSS statistics: And sex and drugs and rock ‘n’ roll* (4th ed.). Los Angeles: Sage, cop.

[CIT0022] Finkelhor D., Hamby S. L., Ormrod R., & Turner H. (2005). The juvenile victimization questionnaire: Reliability, validity, and national norms. *Child Abuse and Neglect*, 29(4), 383–412. 15917079 10.1016/j.chiabu.2004.11.001

[CIT0023] Finkelhor D., Ormrod R. K., Turner H. A., & Hamby S. L. (2005). Measuring poly-victimization using the juvenile victimization questionnaire. *Child Abuse and Neglect*, 29(11), 1297–1312. 16274741 10.1016/j.chiabu.2005.06.005

[CIT0024] Finkelhor D., Turner H., Ormrod R., & Hamby S. L. (2009). Violence, abuse, and crime exposure in a national sample of children and youth. *Pediatrics*, 124(5), 1411–1423. 19805459 10.1542/peds.2009-0467

[CIT0025] Gandubert C., Scali J., Ancelin M. L., Carriere I., Dupuy A. M., Bagnolini G., … Chaudieu I. (2016). Biological and psychological predictors of posttraumatic stress disorder onset and chronicity. A one-year prospective study. *Neurobiol Stress*, 3, 61–67. 27981178 10.1016/j.ynstr.2016.02.002 PMC5146193

[CIT0026] Griffin M. G., Resick P. A., & Mechanic M. B. (1997). Objective assessment of peritraumatic dissociation: Psychophysiological indicators. *American Journal of Psychiatry*, 154(8), 1081–1088. 9247393 10.1176/ajp.154.8.1081 PMC2958429

[CIT0027] Gustafsson P. E., Nilsson D., & Svedin C. G. (2009). Polytraumatization and psychological symptoms in children and adolescents. *European Child and Adolescent Psychiatry*, 18(5), 274–283. 19156354 10.1007/s00787-008-0728-2

[CIT0028] Hawker D. S., & Boulton M. J. (2000). Twenty years’ research on peer victimization and psychosocial maladjustment: A meta-analytic review of cross-sectional studies. *Journal of Child Psychology and Psychiatry and Allied Disciplines*, 41(4), 441–455. 10836674

[CIT0029] Hayes A. F. (2013). *Introduction to mediation, moderation, and conditional process analysis: A regression-based approach*. New York, NY: Guilford Press.

[CIT0030] Hetzel-Riggin M. D., & Roby R. P. (2013). Trauma type and gender effects on PTSD, general distress, and peritraumatic dissociation. *Journal of Loss and Trauma*, 18(1), 41–53.

[CIT0031] Johnson D. M., Pike J. L., & Chard K. M. (2001). Factors predicting PTSD, depression, and dissociative severity in female treatment-seeking childhood sexual abuse survivors. *Child Abuse and Neglect*, 25(1), 179–198. Retrieved from https://www.ncbi.nlm.nih.gov/pubmed/11214810 11214810 10.1016/s0145-2134(00)00225-8

[CIT0032] Koopman C., Classen C., & Spiegel D. (1994). Predictors of posttraumatic stress symptoms among survivors of the Oakland/Berkeley, Calif, Firestorm. *American Journal of Psychiatry*, 151(6), 888–894. 8184999 10.1176/ajp.151.6.888

[CIT0033] Lawyer S. R., Resnick H. S., Galea S., Ahern J., Kilpatrick D. G., & Vlahov D. (2006). Predictors of peritraumatic reactions and PTSD following the September 11th terrorist attacks. *Psychiatry*, 69(2), 130–141. 16822192 10.1521/psyc.2006.69.2.130

[CIT0034] Marmar C. R., Weiss D. S., Metzler T. J., & Delucchi K. (1996). Characteristics of emergency services personnel related to peritraumatic dissociation during critical incident exposure. *American Journal of Psychiatry*, 153(7 Suppl), 94–102. 8659646 10.1176/ajp.153.7.94

[CIT0035] Marmar C. R., Weiss D. S., Schlenger W. E., Fairbank J. A., Jordan B. K., Kulka R. A., & Hough R. L. (1994). Peritraumatic dissociation and posttraumatic stress in male Vietnam theater veterans. *American Journal of Psychiatry*, 151(6), 902–907. 8185001 10.1176/ajp.151.6.902

[CIT0036] McKeever V. M., & Huff M. E. (2003). A diathesis-stress model of posttraumatic stress disorder: Ecological, biological, and residual stress pathways. *Review of General Psychology*, 7(3), 237–250.

[CIT0037] Miller-Graff L. E., Scrafford K., & Rice C. (2016). Conditional and indirect effects of age of first exposure on PTSD symptoms. *Child Abuse and Neglect*, 51(1873–7757 (Electronic)), 303–312. 26427886 10.1016/j.chiabu.2015.09.003

[CIT0038] Nilsson D., Wadsby M., & Svedin C. G. (2008). The psychometric properties of the Trauma Symptom Checklist For Children (TSCC) in a sample of Swedish children. *Child Abuse and Neglect*, 32(6), 627–636. 18584867 10.1016/j.chiabu.2007.09.009

[CIT0039] Ozer E. J., Best S. R., Lipsey T. L., & Weiss D. S. (2003). Predictors of posttraumatic stress disorder and symptoms in adults: A meta-analysis. *Psychological Bulletin*, 129(1), 52–73. Retrieved from https://www.ncbi.nlm.nih.gov/pubmed/12555794 12555794 10.1037/0033-2909.129.1.52

[CIT0040] Sachser C., Keller F., & Goldbeck L. (2017). Complex PTSD as proposed for ICD-11: Validation of a new disorder in children and adolescents and their response to trauma-focused cognitive behavioral therapy. *Journal of Child Psychology and Psychiatry and Allied Disciplines*, 58(2), 160–168. 10.1111/jcpp.12640 27677771

[CIT0041] SALAR (2009). *Levels of local democracy in Sweden*. Stockholm: Swedish Association of Local Authorities and Regions.

[CIT0042] Sayed S., Iacoviello B. M., & Charney D. S. (2015). Risk factors for the development of psychopathology following trauma. *Current Psychiatry Reports*, 17(8), 612. 26206108 10.1007/s11920-015-0612-y

[CIT0043] Shalev A. Y., Peri T., Canetti L., & Schreiber S. (1996). Predictors of PTSD in injured trauma survivors: A prospective study. *American Journal of Psychiatry*, 153(2), 219–225. 8561202 10.1176/ajp.153.2.219

[CIT0044] Steuwe C., Lanius R. A., & Frewen P. A. (2012). Evidence for a dissociative subtype of PTSD by latent profile and confirmatory factor analyses in a civilian sample. *Depression and Anxiety*, 29(8), 689–700. 22653764 10.1002/da.21944

[CIT0045] Sugar J., & Ford J. D. (2012). Peritraumatic reactions and posttraumatic stress disorder in psychiatrically impaired youth. *Journal of Traumatic Stress*, 25(1), 41–49. 22354507 10.1002/jts.21668

[CIT0046] Takizawa R., Maughan B., & Arseneault L. (2014). Adult health outcomes of childhood bullying victimization: Evidence from a five-decade longitudinal British birth cohort. *American Journal of Psychiatry*, 171(7), 777–784. 24743774 10.1176/appi.ajp.2014.13101401

[CIT0047] Tekin A., Karadag H., Suleymanoglu M., Tekin M., Kayran Y., Alpak G., & Sar V. (2016). Prevalence and gender differences in symptomatology of posttraumatic stress disorder and depression among Iraqi Yazidis displaced into Turkey. *European Journal of Psychotraumatology*, 7, 28556. 26886485 10.3402/ejpt.v7.28556 PMC4756623

[CIT0048] The Swedish Ministry of Education and Cultural Affairs (2003). The act concerning the ethical review of research involving humans (2003:460). Stockholm: The Swedish Ministry of Education and Cultural Affairs.

[CIT0049] The Swedish National Agency for Education (2007). [Data set]. Retrieved from http://siris.skolverket.se/

[CIT0050] Trickett P. K., Noll J. G., & Putnam F. W. (2011). The impact of sexual abuse on female development: Lessons from a multigenerational, longitudinal research study. *Development and Psychopathology*, 23(2), 453–476. 23786689 10.1017/S0954579411000174 PMC3693773

[CIT0051] U.S. Department of Health & Human Services (2016). Child maltreatment 2014. Retrieved from http://www.acf.hhs.gov/

[CIT0052] United Nations Division for Sustainable Development (2015). *Transforming our world: The 2030 agenda for sustainable development*. Australia: Author.

[CIT0053] United Nations Office of the High Commissioner for Human Rights (2000). Protocol to Prevent, Suppress and Punish Trafficking in Persons, Especially Women and Children, Supplementing the United Nations Convention against Transnational Organized Crime 55/25. World Health Organization (WHO).

[CIT0054] Werner K. B., & Griffin M. G. (2012). Peritraumatic and persistent dissociation as predictors of PTSD symptoms in a female cohort. *Journal of Traumatic Stress*, 25(4), 401–407. 22833467 10.1002/jts.21725 PMC4112724

[CIT0055] Zona K., & Milan S. (2011). Gender differences in the longitudinal impact of exposure to violence on mental health in urban youth. *Journal of Youth and Adolescence*, 40(12), 1674–1690. 21400207 10.1007/s10964-011-9649-3

[CIT0056] Zwi A. B., Krug E. G., Mercy J. A., & Dahlberg L. L. (2002). World report on violence and health–exploring Australian responses. *Australian and New Zealand Journal of Public Health*, 26(5), 405–408. Retrieved from https://www.ncbi.nlm.nih.gov/pubmed/12413278 12413278 10.1111/j.1467-842x.2002.tb00334.x

